# Editorial: Recent Advances in In Vitro and In Vivo Multi-Omics Analyses of Extracellular Vesicles: Therapeutic Targets and Biomarkers

**DOI:** 10.3389/fmolb.2021.784436

**Published:** 2021-10-27

**Authors:** Marcello Manfredi, Evan Williams, William C. Cho, Marco Falasca

**Affiliations:** ^1^ Department of Translational Medicine, University of Piemonte Orientale, Novara, Italy; ^2^ Center for Translational Research on Autoimmune and Allergic Diseases, University of Piemonte Orientale, Novara, Italy; ^3^ Luxembourg Centre for Systems Biomedicine, Université du Luxembourg, Luxembourg, Luxembourg; ^4^ Department of Clinical Oncology, Queen Elizabeth Hospital, Kowloon, Hong Kong SAR, China; ^5^ Metabolic Signalling Group, Curtin Medical School, Curtin Health Innovation Research Institute, Curtin University, Perth, WA, Australia

**Keywords:** extracellular vesicles, exosomes, mass spectrometry, biomarkers, therapeutic targets

Extracellular vesicles (EVs) are membrane-surrounded nano-sized vesicles released by cells. EVs are delivered in a biologically active form to the recipient cells, playing important roles in cell-cell communications via cross-transferring of important signaling components (proteins, lipids, and RNAs, etc.) in physiological and pathological conditions. While their biological functions are not completely clear, they were used not only to discover new potential biomarkers and therapeutic targets, but also to understand the mechanisms involved in the development of diseases.

In the current Research Topic (RT), we provided recent, novel, and mechanistic insights into the role of EVs in diseases such as cancer, viral infection, etc. Original articles and reviews that focused on EVs to study therapeutic approaches, biomarkers, and mechanisms involved in diseases are rigorously reviewed and being selected.

In recent years, mass spectrometry (MS) characterization of isolated EV molecules has gathered interest due to the high sensitivity and small initial sample volumes required for MS analysis. In addition, thanks to the widespread untargeted approaches, comprehensive and deep proteomic, metabolomic and lipidomic profiles are widely used to improve the understanding of the biological role of EVs. In this RT, new evidence regarding the role of EVs in multiple diseases and their biological functions is presented.

It has been already demonstrated that the promising use of exosomes for therapeutic applications. In this RT, Houang et al. studied the regenerative potentials for cutaneous tissue by exosomes released from primary mesenchymal stem cells (MSCs) originated from the bone marrow (BM), adipose tissue (AD), and umbilical cord (UC) under serum- and xeno-free condition. The authors showed that MSC-derived exosomes can regulate several biological events associated with wound healing processes and that BMMSCs and UCMSCs under clinical condition secreted exosomes are promising to develop into therapeutic products for wound healing treatment.

Study how EVs are released by cells could also open new avenues for innovative therapeutic approaches against cancer. In glioma, tumor-derived EVs have been shown to support progression via several mechanisms. van Solinge et al. evaluated how Ras-associated protein 27a (Rab27a) affects EVs release of small EVs from glioma cells. They found that the knockdown of Rab27a in GL261 glioma cells decreased the release of small EVs, but not the release of larger EVs, while there was no difference in tumor growth or overall survival in the *in vivo* model, suggesting that future studies should be cautious in using the knockdown of Rab27a as a means of studying the role of small EVs in glioma growth.

Interestingly, exosomes are also released during viral infection. It was found that host cells release EVs carrying viral and host components that can modulate the immune response. Barberis et al. investigated how SARS-CoV-2 infection modulates exosome content, exosomes’ involvement in disease progression, and the potential use of plasma exosomes as biomarkers of the disease severity. They analyzed circulating exosomal proteins from COVID-19 patients and identified several molecules (C1QB, C1QC, C1R, C1S, C2, C3, C4A/C4B, C4BPA, C4BPB, C5, C6, C8A, C8G, CFI, CR1, MBL2, SERPING1) involved in the immune response, inflammation, and activation of the coagulation and complement pathways. In addition, they found several potential diagnostic biomarkers such as fibrinogen, fibronectin, complement C1r subcomponent, and serum amyloid P-component. Importantly, the authors identified the presence of SARS-CoV-2 RNA in the exosomal cargo, which suggests that the virus might use the endocytosis route to disseminate the infection.

On the other hand, the content of EVs is dynamic, which reflects the individual daily habits and fasting-fed state metabolic characteristics. Zhou et al. discussed the role of adipocyte-derived EVs in the regulation of whole-body metabolism under physiological and pathophysiological conditions. In fact, adipose tissue may be a major source of circulating exosomal miRNAs that not only may regulate metabolic homeostasis, but could also promote insulin resistance in other organs. In addition, adipocyte-derived miRNAs in EVs may also induce obesity-related changes and it may affect the biological behaviors of surrounding tumor cells.

The study of EVs and their content are vital to better understand the mechanisms behind cell-cell communication, disease development, and physiological conditions. We envision the use of multi-omics techniques such as proteomics, metabolomics, lipidomics, genomics, and transcriptomics will be valuable to get a clearer and a holistic view of the biological processes that are driven by EVs.

